# Left Vertebral Artery Arising Directly From the Arch of the Aorta With a Prevailing Vascular Problem: A Case Report

**DOI:** 10.7759/cureus.51591

**Published:** 2024-01-03

**Authors:** Magda A Eldomiaty, Mostafa M Elnaggar, Emad A Albadawi

**Affiliations:** 1 Anatomy, Embryology, and Histology, College of Medicine, Al-Rayan Colleges, Al-Madinah, SAU; 2 General Surgery, Elmanial Specialized Hospital, Cairo University Hospitals, Cairo, EGY; 3 Vascular Surgery, Helwan University, Cairo, EGY; 4 Anatomy, College of Medicine, Taibah University, Al-Madinah, SAU

**Keywords:** left common carotid artery, abnormal origin, left vertebral artery, brachiocephalic trunk, arch of aorta

## Abstract

Aberrant origin of the vertebral artery is a rare case. Due to its important clinical significance during operations in the superior mediastinum and the root of the neck, the variations of this artery should be clarified, and any cadaveric case should be explored specifically if accompanied by any vascular problem. In this cadaveric case, the embalmed male cadaver was found to have a pacemaker wire inserted in the heart through the superior vena cava, denoting a vascular incompetence due to sinus arrhythmia. The left vertebral artery was found to originate from the aortic arch, positioned between the left common carotid artery and the left subclavian artery. It traveled upward behind the left common carotid artery, passing in front of the stellate ganglion and the ventral rami of cervical spinal nerves before entering the left foramen transversarium of the C6 vertebra. This atypical left vertebral artery, which had an unusual origin from the arch of aorta, was distinct from the right vertebral artery, that typically arises from the right subclavian artery. Also, the left atypical artery was found to be narrower and longer than the right one. Additionally, the left common carotid artery exhibited an unusual origin from the beginning of the brachiocephalic trunk. The present case report would be of significance for vascular surgeons in designing surgical intervention in the root of the neck and for clinicians responsible for monitoring patients with variant vertebral arteries to effectively manage potential vascular complications.

## Introduction

The vertebral artery (VA) is the first and largest branch of the subclavian artery (SCA). It originates most often from the posterosuperior aspect of the SCA and less frequently from its anterior aspect. Normally, the SCA develops from the persistent seventh intersegmental artery and includes the origin of VA. The origin of VAs from the aorta might be due to the increased absorption of the left SCA between the origin of the aortic arch and the VA or due to the formation of a part of the aortic arch from the left 7th intersegmental artery [1,2].

The course of VA is divided into three segments before entering the cranial cavity to unite with the VA of the other side to form the basilar artery and take its vital part in the blood supply of the brainstem and cerebellum [3,4]. The first segment (the ostial segment) can be easily identified from other branches of the SCA, as it gives no branches. It ascends in the vertebral triangle of the neck extending from its origin at the SCA till it enters into the lowest transverse foramen, usually at the C6 level, but sometimes it enters the transverse foramen of the fifth, fourth, or seventh cervical vertebrae. In the vertebral triangle, it passes behind the internal jugular and vertebral veins and is crossed by the inferior thyroid artery and the thoracic duct. It then reaches the transverse process of the seventh cervical vertebra, with the stellate ganglion located at its anteromedial border at the level of the first costovertebral junction [3,5].

Anomalous origin and course, duplication, tortuosity, kinking, and aneurysmal formation are anomalies of the VA that are commonly associated with cerebrovascular events. The anomalous origin of the VA is a rare variation that warrants thorough investigation, especially before performing procedures involving the carotid artery, aortic arch, or esophagus surgery. This is especially crucial in cases of "vertebral arteria lusoria," as lack of knowledge of this aberrant VA could potentially lead to life-threatening situations [3,6].

Exploring cadaveric cases of different aberrant origins of VAs especially when accompanied by other vascular anomalies or apparent health problem in the cadavers is of extreme importance for diverting attention to clinical implications of such cases.

## Case presentation

**Figure 1:**
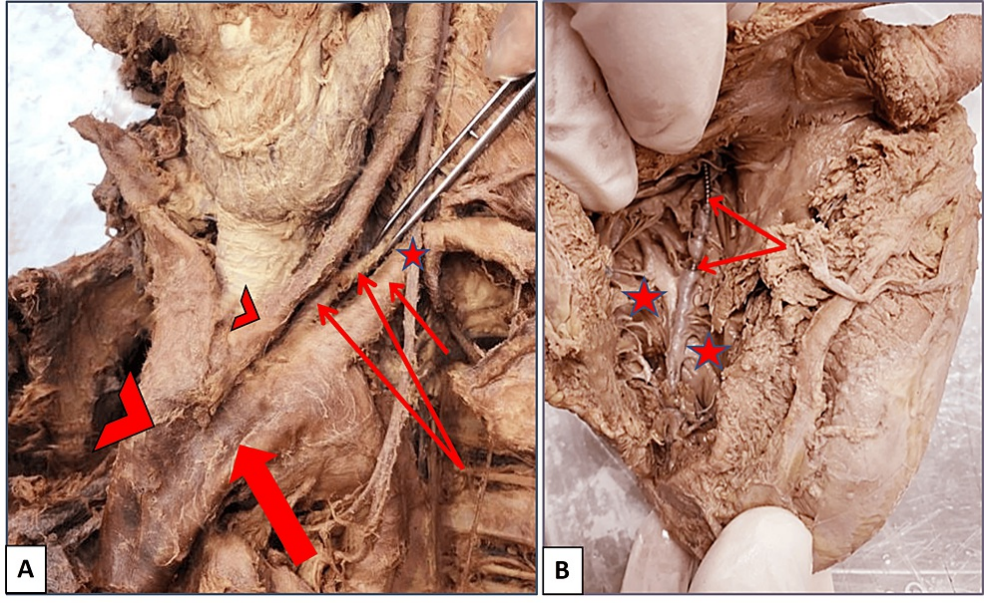
Photographs of the superior mediastinum with the root of the neck and the interior of the heart of a donated embalmed male cadaver around 50 years old. (A) The left vertebral artery (double thin arrow) arising abnormally from the arch of the aorta (thick arrow), the left common carotid artery (small arrowhead) arising from the beginning of the brachiocephalic trunk (large arrowhead), and the left subclavian artery (thin short arrow) and the left vagus nerve (star). (B) The cavity of the right ventricle (stars) and the wire of the inserted pacemaker (double thin arrow).

During routine dissection of the thoracic cavity of a donated embalmed male cadaver around 50 years old in the Department of Anatomy, Taibah Medical College, Al-Madinah Al-Munawwarah, Saudi Arabia, we observed a pacemaker under the skin of the thoracic cage with its wire extended inside the thoracic cavity. On opening the thoracic cavity and exploring the structures in the superior mediastinum, we observed a rare case of an extra branch originated from the upper surface of the aortic arch between the origin of the left common carotid artery and left SCA. When tracing this branch in the neck, it was observed that it was the left VA, which had an anomalous origin from the aortic arch. It ascended behind the left common carotid artery, with the stellate ganglion and ventral rami of cervical spinal nerves situated posterior to it. It entered the left foramen transversarium of the C6 vertebra (Figure 1A). In the same case, the left common carotid artery was found to arise from the beginning of the brachiocephalic trunk instead of arising from the arch of the aorta itself (Figure 1A). The right VA was found to originate from the posterior aspect of the first part of the right SCA, pass medially behind the common carotid artery, and enter the right foramen transversarium of the C6 vertebra. The prevertebral segments of both the right and left VAs were examined from the origin to the foramina transversaria of the cervical vertebra. Both VAs were assessed morphometrically using a Vernier caliper for both the diameter at the origin of the artery and the length of the prevertebral segment from the origin till entry into the transversarium foramen. The abnormal left VA was found to be narrower and longer (3.6 and 74 mm, respectively) as compared to the right VA (11 and 40 mm, respectively). Notably, we traced the wire of the pacemaker till the left brachiocephalic vein, superior vena cava, right atrium, and right ventricle of the heart to detect any other vascular abnormality. Photographs were captured to document the concurrent vascular complications associated with the variant origin of the VA, as shown in Figure 1B.

## Discussion

An atypical origin of the left VA from the aortic arch is recorded in approximately 6% of the population [7]. The direct aortic origin of the left VA is the most frequent variant origin, representing 2.4-5.8% from many large autopsy groups [8]. Among patients who underwent cerebral angiography for various reasons, the direct origin of the left VA from the aortic arch was observed in approximately 2.4-2.5% of cases [9,10], while in patients with expected extracranial cerebrovascular disorder, it was observed in 5.25% [2,11]. Its direct origin from the aorta between the left common carotid artery and left SCA was found in 4.1% of patients having cerebrovascular diseases [12]. Other variations of the origin of VA were similarly described from the thyrocervical and brachiocephalic trunks and from the common carotid and external carotid arteries [13].

In the present case, the left VA arose abnormally from the arch of the aorta between the left common carotid artery and left SCA. This was accompanied by the anomalous origin of the left common carotid artery from the beginning of the brachiocephalic trunk. This case was associated with cardiovascular problems as a pacemaker was inserted into the right ventricle of the heart. As observed in the current case, the direct aortic arch origin of the left VA has been linked to vascular issues such as the presence of the retroesophageal right SCA with the thoracic duct terminating on the right side [14]. Also, there is significantly increased risk of serious complications in the VA, including dissection, aneurysm, critical stenosis, and thrombosis [2,10].

Abtahi et al. reported a higher incidence of aberrant origin of the left VA than the right one. They documented that many cases of aberrant origin of the VA were asymptomatic and that only 5.5% of the patients presented with vascular complaints and their symptoms were probably related to this aberrant origin [15].

In our case, the left VA, which had an aberrant origin, had a significantly smaller diameter compared to the normally originating right VA. Also, the length of the prevertebral segments of the variant artery was much longer than the normal right VA. These variations in width and length of the variant artery were the same as those reported by Sawant and Panicker et al. [7,16], and they can explain the increased incidence of this variation in cerebrovascular disease [12].

Also, in this case, the left VA, which originated from the aortic arch, entered the left foramen transversarium at the C6 level. The location where the aberrant left VA enters the foramina transversaria can vary, but it typically does so at the C5 level rather than the C6 level [17]. The concomitant presence of variant left VA originating from the aortic arch and left common carotid artery originating from the beginning of the brachiocephalic trunk might be of high significance for the associated vascular disorders [7,14].

## Conclusions

This case underscores the significance of prioritizing thorough preoperative assessment for patients undergoing neck and chest surgeries, emphasizing the need for meticulous screening to detect the possible existence of an unusual origin of the VA. Moreover, this case underscores the importance of closely monitoring patients who are found to have variant VAs during cerebral angiography performed for various purposes. This caution is essential to proactively address potential complications associated with the abnormal VA.
